# Neural and behavioural correlates of repeated social defeat

**DOI:** 10.1038/s41598-018-25160-x

**Published:** 2018-05-01

**Authors:** Julie M. Butler, Sarah M. Whitlow, David A. Roberts, Karen P. Maruska

**Affiliations:** Department of Biological Sciences, Louisiana State University, 202 Life Sciences Bldg., Baton Rouge, LA 70803 USA

## Abstract

Dominance hierarchies are common across the animal kingdom and have important consequences for reproduction and survival. Animals of lower social status cope with repeated social defeat using proactive and reactive behaviours. However, there remains a paucity of information on how an individual’s coping behaviours changes over time or what neural mechanisms are involved. We used a resident-intruder paradigm in the African cichlid fish *Astatotilapia burtoni* to investigate the neural correlates of these two opposing behaviour groups. Fish initially used both proactive and reactive behaviours, but had a dramatic increase in use of proactive behaviours during the third interaction, and this was followed by cessation of proactive behaviours and exclusive use of reactive coping. By quantifying neural activation in socially-relevant brain regions, we identify a subset of brain nuclei, including those homologous to the mammalian amygdala, showing higher activation in fish displaying proactive but not reactive behaviours. Fish displaying reactive behaviours had greater neural activation in the superior raphe, suggesting a possible conserved function during social defeat across vertebrates. These data provide the first evidence on the involvement of specific brain regions underlying proactive and reactive coping in fishes, indicating that these nuclei have conserved functions during social defeat across taxa.

## Introduction

The ability of all animals to adequately detect and respond to a stressful situation is necessary for survival. Biotic (e.g. predators), abiotic (e.g. temperature fluctuations, anthropogenic noise), and social (e.g. aggression) stressors are part of everyday life, and how an animal responds to these stressors can vary greatly from individual to individual^1–3^. Generally, animals adopt one of two coping styles that are defined by the individuals’ behavioural and physiological responses to stress^4,5^. Proactive animals are said to be more “bold”, respond with aggression, or attempt to escape the stressor^4,6^. In addition, they are characterized by lower stress-induced corticosteroid levels, lower brain serotonin levels, and higher brain dopamine levels. Conversely, reactive animals are described as “shy”, do not attempt to escape the stressor, have high hypothalamic-pituitary-adrenal (HPA) axis reactivity and brain serotonin levels, and low brain dopamine levels^2,6–10^.

Social hierarchies are found throughout the animal kingdom and have important consequences for reproduction and survival^11,12^. These hierarchies typically result from repeated social interactions with the same individuals where animals of lower social rank are constantly suppressed by animals of higher rank, but can be maintained by dominant individuals interacting with only a few individuals of lower rank^13–15^. In response to an acute social stress (i.e. single social defeat), animals adopt initial coping behaviours. It remains unknown, however, whether animals may change their coping behaviours when repeatedly exposed to the same social stressor. As with other stressors, does an animal habituate to a social stressor when it may not be advantageous to always respond in a proactive manner?

Due to their low maintenance, relatively quick development, and ease of genetic manipulations, fishes are commonly used as model animals for studying neural underpinnings of neurological and psychological disorders, such as anxiety and depression^16^. The behavioural and physiological responses of fishes to both social and environmental stressors are well documented^6,9,17–28^. Although recent research has begun to untangle the neural mechanisms underlying proactive and reactive coping^9^, there remains a lack of information on the neural circuits regulating these complex behaviours. If fishes are to be used as appropriate models for neuropsychological studies, we must first understand which brain regions are involved in regulating these two opposing coping strategies.

Immediate early genes (IEGs) and other markers of neural activation are useful tools for measuring region-specific brain activation in neuroethological studies. Expression of IEGs, like the transcription factors *cfos* and *egr1*, correspond to the changes in gene expression levels caused by extracellular signals. They are commonly used to compare neural activation patterns in fishes and other vertebrate taxa under different social or sensory contexts to evaluate where this information is processed in the brain^29–31^. Although not an IEG, immunohistochemistry for phospho-S6 ribosomal protein (pS6) stains ribosomal proteins that have been phosphorylated in the previous ~1 hour. This increased phosphorylation is tied to increased translation, and thus pS6 is emerging as another useful marker for the neural activation toolkit^32–34^.

The African cichlid fish *Astatotilapia burtoni* is an emerging model system in behavioural neuroscience due to their well characterized behaviours, physiology, and neuroendocrine systems^35–37^. Males exist as two distinct phenotypes, dominant and subordinate, which they can rapidly and reversibly switch between. Dominant males are characterized by their territoriality and high level of aggression. When ascending or descending in social rank, a suite of well-documented behavioural, physiological, and neurobiological changes occur^38–40^. Their dynamic social interactions means that males are constantly challenging each other’s status to acquire a territory and their relative position in the hierarchy can change frequently. Because of this, they are an excellent system to investigate the behavioural and neural correlates of repeated social defeat.

We used a resident-intruder paradigm to expose male African cichlid fish, *Astatotilapia burtoni*, to repeated social defeat from the same aggressor. By allowing the intruders to be socially-defeated for up to 5 consecutive days, we observed that most *A. burtoni* males initially perform both proactive and reactive behaviours. However, their use of proactive behaviours increases until they reach a threshold, most commonly on the third interaction, and then switch to reactive coping behaviours. By comparing activation in 14 different brain regions of fish that were not defeated and defeated two, three, and four times, we describe a novel neural circuit regulating the use of proactive and reactive coping behaviours. These data provide invaluable insight into the conservation of neural networks regulating coping behaviours across vertebrates that will be relevant to validating the use of fishes and other model organisms for understanding the neural correlates of neurological and psychological conditions.

## Methods

### Experimental animals

Laboratory-bred *Astatotilapia burtoni* were maintained in an environment that resembled their natural habitat (28–30 °C; 12 h L:12 h D cycle, full spectrum illumination; pH = 8.0; conductivity: 300–500 µm/cm). Fish were housed in 113.56 L aquaria and fed cichlid flakes once daily and supplemented with brine shrimp twice weekly. Communities of juvenile fish were placed in 37.85 L aquaria containing gravel covered bottoms and a single half terracotta pot to serve as a territory. Experimental male fish were selected shortly after the onset of adult-typical coloration and social behaviours (~60 days of age). Only yellow-morph males were used as experimental animals. In addition, all fish were the largest fish in their community prior to selection and no other fish had challenged their dominance status (standard length: 35.557 mm ± 0.627 mm; body mass: 1.283 g ± 0.054 g; gonadosomatic index: 0.646 ± 0.045). This ensured that no experimental fish had previously experienced prolonged social conflict and that all animals had a similar social background. As such, subordinate individuals (based on behaviour and appearance) were excluded. Further, subject fish acclimated to the experimental tank for 2 days (see below) so subordinate individuals would have risen to dominance within a few hours of being placed in the acclimation tank (dominant phenotype is the default in this species). All experiments were performed in accordance with the recommendations and guidelines stated in the National Institutes of Health (NIH) Guide for the Care and Use of Laboratory Animals, 2011. The experimental protocol was approved by the Institutional Animal Care and Use Committee (IACUC) at Louisiana State University, Baton Rouge, LA.

### Experimental set up and behavioural analysis

**Figure 1:**
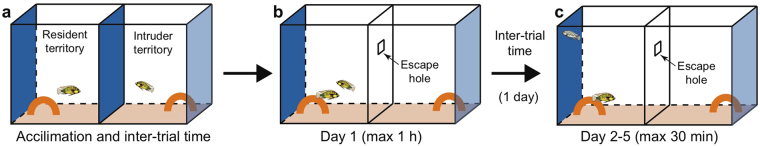
A resident-intruder paradigm was used to induce repeated social defeat in *Astatotilapia burtoni* males. (**a**) A 37.85 L tank was divided in two compartments by an opaque blue barrier, and views of neighbouring tanks were obstructed. One fish was allowed to acclimate to each territory for 2 days. (**b**) On the morning of the trial, the blue barrier was removed between the two compartments revealing a clear barrier with a small escape hole. The intruder was quickly netted from his home territory and placed into the resident’s territory and allowed to interact until he either escaped through the hole or 1 hour had passed. (**c**) On subsequent trials (days 2–5), the intruder was quickly netted from his territory and placed into the resident’s compartment, where he quickly faded his stereotypical “dominant” coloration. These trials continued until either the intruder escaped or 30 minutes had passed. At the conclusion of all trials the intruder was placed back into his home territory and the blue barrier was replaced (as shown in a) until the subsequent trial.

To examine how *A. burtoni* males respond to repeated social defeat, we used a resident-intruder paradigm. A single 37.85 litre tank (50.8 × 31.12 × 25.4 cm) was divided into two equal compartments by a clear acrylic barrier containing an “escape hole” (2.0 × 1.5 cm) located 20 cm from the bottom of the tank (Fig. 1). Each compartment contained a halved terracotta pot that was angled toward the barrier so the inside was visible to the other compartment. Dominant resident fish were chosen based on their territorial displays in community tanks for at least one week, used for only 1–2 experiments, and were ~10% larger than the experimental fish (i.e. intruder). This ensured that the subject resident fish would win the initial territorial interaction and the intruder would be defeated. All fish were acclimated for ~48 hours during which time they were visually isolated from each other and neighbouring tanks using an opaque blue, acrylic barrier.

On the morning of trials (10 am–12 pm), fish acclimated to the video camera placed in front of the tank for approximately 5 min. The opaque barrier was then removed while leaving the clear barrier with the escape hole in place, and the intruder was quickly netted from his home territory and moved to the resident’s compartment. The trial ended when the intruder escaped through the hole or a maximum time limit was reached (see below), at which time the intruder was placed back into his compartment and the opaque blue barrier replaced.

To allow for repeated acute social defeat, intruders were exposed to the same resident once daily for 2, 3, 4, or 5 days in row. This paradigm allowed for an initial territorial fight to occur on Day 1 (max time = 1 hour). By separating trials by one day, the intruder could re-establish his dominance in his own compartment. During subsequent trials (max time = 30 min), the intruder was immediately suppressed by the resident without a true territorial fight occurring (fight criteria based on^41^). This paradigm most closely resembles the natural ecology of the Lake Tanganyikan lek system in which males can be exposed and supressed by the same individuals on a regular basis^36^.

All trials were video recorded and stereotypical aggressive behaviours^35,41^ were quantified for both the resident and intruder fish by independent observers blind to the trial identity. In addition to aggressive behaviours, we quantified “searching” and “hiding/freezing” behaviours performed by the intruder. Searching was defined as swimming perpendicular into the wall of the tank or barrier, often in the vertical plane, and had to last a minimum of two seconds. Freezing (or hiding) was defined as the intruder remaining stationary in the bottom corner of the tank or at the top of the water column, typical submissive behaviours. To account for variation in the resident’s aggression across trials, we further quantified how the intruder responded to an attack from the resident. The responses were divided into 5 categories: no response (stationary, no attempt to flee), flinch, flee to hide/freeze, flee to search, and aggression. For full definitions and criteria of all behaviours, see Supplemental Information. Using a principal component analysis (see Statistics section for details), we classified behaviours as proactive or reactive. Behaviours positively loaded in component 2 were designated at proactive behaviours while those negatively loaded were deemed reactive behaviours.

Control animals acclimated to the home intruder compartment for 48 hours before being moved into the resident’s compartment sans resident. This controlled for the stress associated with social isolation during acclimation, handling, and transfer to a novel territory, and allowed us to be sure that behaviour and brain differences were due to social defeat and not stress related to the behavioural paradigm itself. Animals were not collected following the initial territorial defeat because previous studies in *A. burtoni* have already characterized the neural correlates of social decent and territory loss^30^.

Ninety-six total animals were used in this experiment, eight of which were control experiments (no resident). Fish were removed for technical reasons (N = 18), if the intruder won the initial fight (N = 3), or if the resident swam through the escape hole (N = 10). The remaining 57 animals were analysed as described above.

### Tissue collection and preparation

To compare brain activation patterns of fish displaying either proactive or reactive behaviours observed on the various days of repeated social defeat, we collected brains from fish that had suffered 2, 3, or 4 bouts of defeat to the same aggressor. All fish collected on day 2 were confirmed to display equal levels of proactive and reactive behaviours; whereas, fish collected on days 3 and 4 used predominately proactive and reactive behaviours, respectively (see Results for classification details). Animals were collected 30 minutes after the conclusion of the trial to allow for expression of the pS6 neural activation maker. If the intruder swam through the escape hole, they were collected 30 minutes after their escape. If fish failed to escape, they remained in the resident’s compartment for the additional 30 minutes until collection. Fish were anesthetized in ice cold fish water, measured for standard length (SL) and body mass (BM), and killed by rapid cervical transection. Gonads were removed and weighed (gonad mass, GM) to calculate gonadosomatic index (GSI = (GM/BM) × 100). Brains were exposed and fixed in the head overnight at 4 **°**C in 4% paraformaldehyde (PFA) in 1x phosphate-buffered saline (1xPBS), rinsed for 24 hours in 1xPBS, and cryoprotected overnight in 30% sucrose in 1xPBS. Brains were then embedded in OCT media, sectioned in the transverse plane on a Thermo Scientific™ HM525 NX cryostat at 20 µm, and collected onto 3 alternate sets of charged slides. Slides were dried flat at room temperature for 2 days prior to storage at −80 °C.

### Immunohistochemistry for pS6

**Figure 2:**
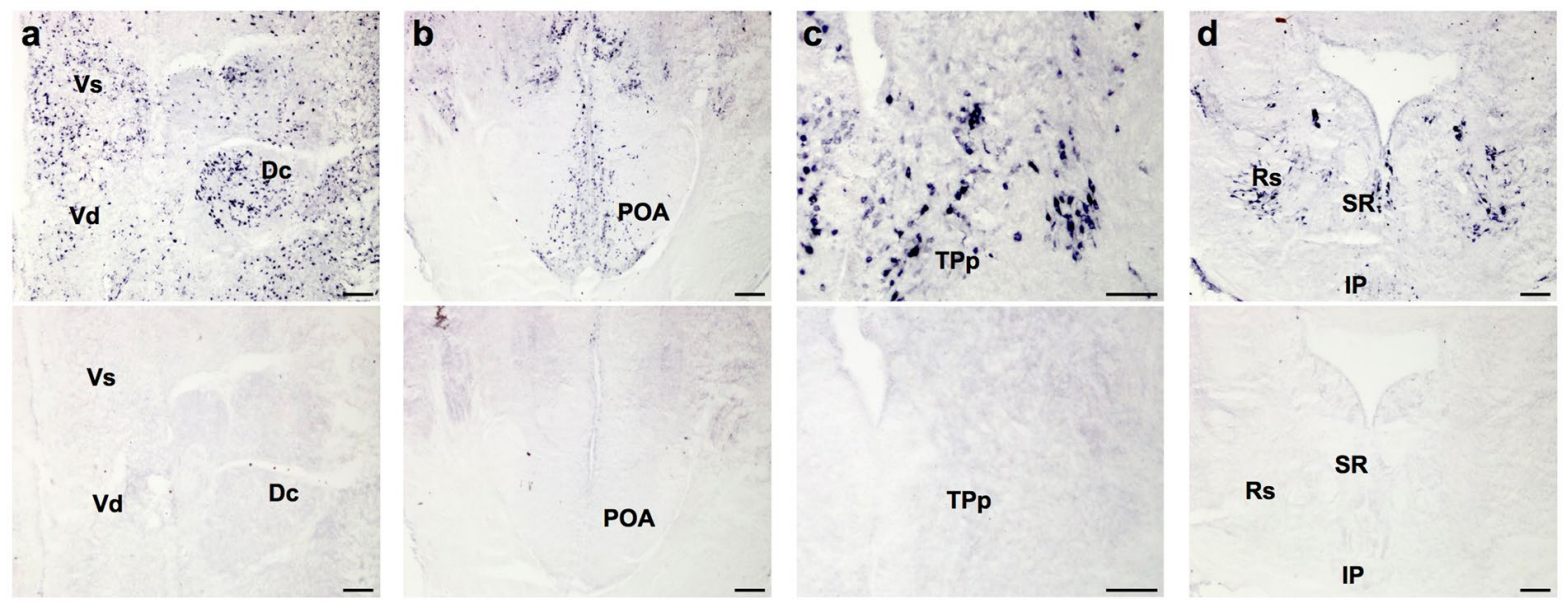
Representative coronal sections in different brain regions showing pS6 antibody specificity. Positive pS6 staining produces clear distinctly stained cells throughout many brain regions (top row), but staining is completely eliminated when the antibody is pre-absorbed with a pS6 blocking peptide on adjacent sections (bottom row). See text for abbreviations. Scale bars represent 100 µm in (**a**,**b** and **d**), and 50 µm in (**c**).

Sectioned brains were stained with the neural activation marker phospho-S6 ribosomal protein (pS6). Slides were brought to room temperature and tissue was outlined with a hydrophobic barrier before being rinsed in 1xPBS. Nonspecific binding was blocked by incubating slides in 1xPBS containing 0.2% bovine serum albumin (BSA), 0.3% triton-X, and 5% normal goat serum (NGS) for 2 hours prior to incubation in pS6 primary antibody (1:1500; prepared in blocking solution; Cell Signalling pS6 ribosomal protein S235/236 antibody) overnight at 4 **°**C. Slides were then rinsed in 1xPBS, incubated in biotinylated goat anti-rabbit secondary antibody (Vector Labs; 1:277; prepared in 1xPBS with 5% NGS) for 2 hours at RT, rinsed in 1xPBS, quenched with 1.5% H_2_O_2_ for 8 minutes, rinsed in 1xPBS, incubated with Vectastain ABC prepared in 1xPBS for 2 hours, and rinsed in 1xPBS. Staining was then visualized by reaction with DAB for ~30 minutes, rinsed in DI water, dehydrated in an alcohol series, cleared in xylene, and coverslipped with Cytoseal-60. To verify antibody specificity, a pre-absorption control was run simultaneously on an alternate set of sectioned brain slides and showed no reaction product (Fig. 2).

### Quantification of brain activation

Quantification of pS6 staining was done as previously described for *cfos* staining^29,42^. Briefly, slides were visualized on a Nikon Eclipse Ni microscope, images were taken at the highest magnification that encompassed the entire brain area of interest, and borders and gridlines were applied. Three to five boxes per section were quantified, and cell density was calculated by dividing the number of cells by the area of the quantified boxes. Three to four consecutive sections were quantified for each region and averaged together for a cell density value of that region in a particular animal. Brightfield and phase contrast was used to visualize cytoarchitecture and brain nuclei in relation to pS6-labeled cells. A cresyl violet stained *A. burtoni* reference brain, *A. burtoni* brain atlas, and other relevant papers^40,43–45^ were used for identification of neuroanatomical markers.

We quantified activation in 14 brain regions, including but not limited to the social decision making network (as defined in^46^). Additional regions were selected based on their known role in social defeat in other taxa (i.e. superior raphe)^6,47,48^, or localization of stress-related neuropeptide-expressing cells (i.e. corticotrophin releasing factor and urotensin1-expressing cells in the Dc^49^; JMB personal observation). Some SDMN regions (i.e. PAG, VTn) were not included due to their small size and lack of reliable neuroanatomical and stereotactical markers for consistent identification and quantification.

### Statistical analysis

All raw data and detailed protocols are available upon request. Data were analysed in SPSS 24 and SigmaPlot 12.3. We used a principal component analysis to classify behaviours as proactive or reactive. Missing values (i.e. outliers determined via Iglewicz and Hoaglin’s test for multiple outliers with a Z = 3.5) were excluded listwise and components 1 and 2 were plotted in rotated space (varimax rotation) for ease of visualization.

Neural activation levels (i.e. pS6-stained cell density) of individual regions were compared using an ANOVA followed by Tukey’s test for post-hoc comparisons. We chose not to use corrections for multiple testing because Bonferroni and similar procedures reduce statistical power and increase the risk of type II errors, especially in small sample sizes^50^. To further visualize differences in neural activation patterns in animals displaying different coping behaviours, we used heatmaps based on Pearson correlation coefficient values and a discriminate function analysis as done previously^29^. All 14 brain regions were used in the DFA, all groups were considered equal, and classification was done using within group covariance.

## Results

### Behaviour

A total of 96 experimental animals were used for this experiment. Of these, 18 were removed for technical reasons (water level too low, water temperature too low or high, etc), and eight were control animals (no resident, see methods for details). Of the remaining 70 trials, the intruder won the fight three times, and the resident swam through the escape hole ten times. The remaining 57 animals were analysed as described above. In nine of these trials, the intruder hid in the corner the entire trial duration on all days, was deemed a “non-responder”, and also removed from further analyses. Thus, the remaining 48 experimental animals were used for behaviour analyses described below.

**Figure 3:**
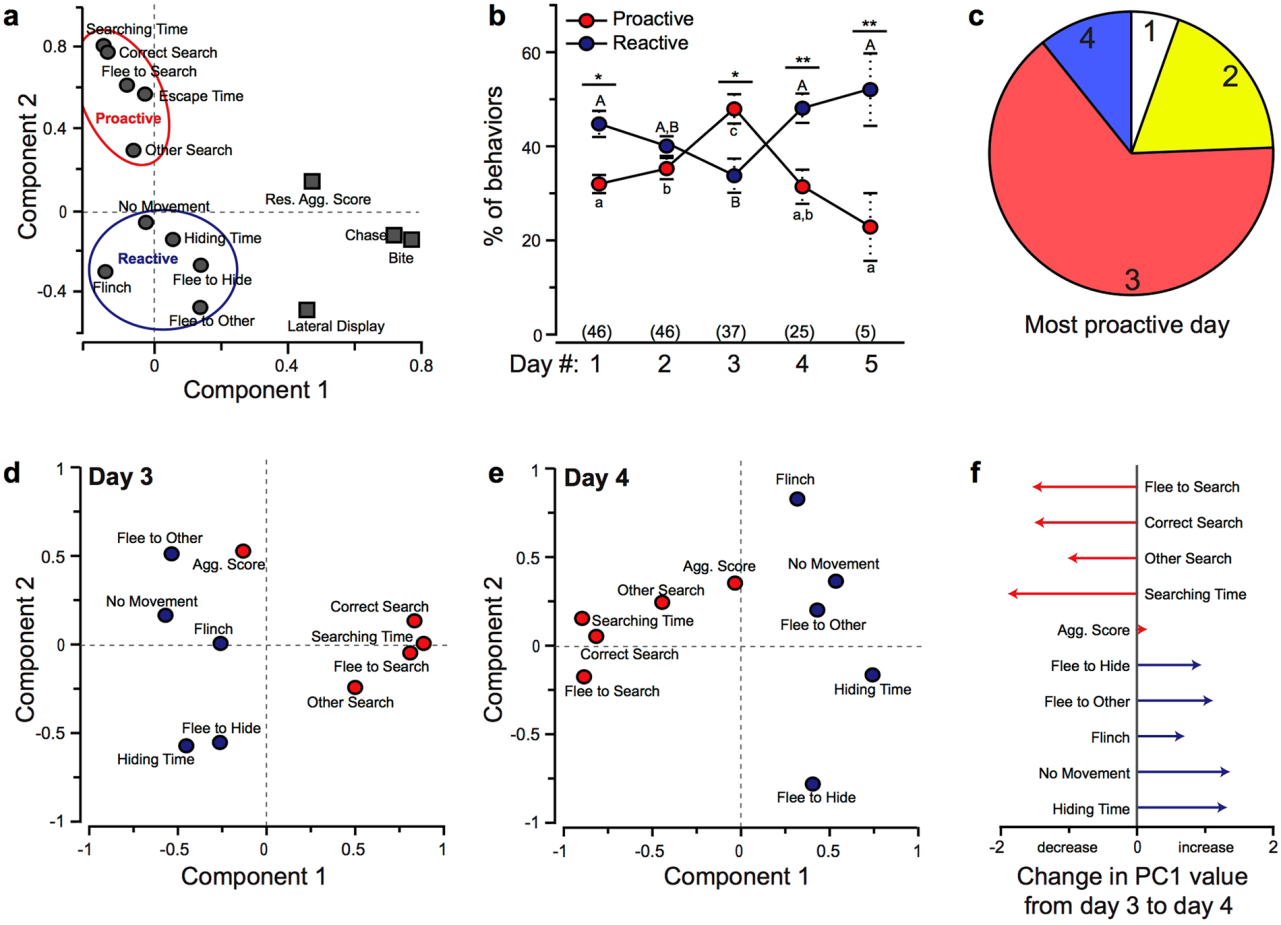
*Astatotilapia burtoni* males switch between reactive and proactive coping behaviours following repeated social defeat from the same aggressor. (**a**) A principal component analysis was used to classify behaviours as either proactive or reactive. Component 1 primarily distinguished resident behaviours (squares) while component 2 was related to intruder behaviours (circles). Behaviours with a positive loading value on component 2 were classified as proactive while negatively loaded values were classified as reactive. (**b**) Use of reactive and proactive coping behaviours changes with the number of defeat interactions. Proactive behaviours (red) peak on day 3 of repeated social defeat, but fish quickly return to using reactive behaviours (blue). Numbers in parentheses represent sample size. Different upper- and lower-case letters indicate differences in reactive and proactive behaviours, respectively, across days. Asterisks indicate difference between proactive and reactive behaviours each day. (**c**) The majority of animals had their most proactive trial occur on day 3, but ~30% were most proactive on days 1, 2, or 4 of repeated social defeat. (**d**,**e**) A PCA of intruder behaviours on day 3 (**d**) and day 4 (**e**) of repeated social defeat represents the relative use of each behaviour, where more frequently used behaviours load positively into PC1. (**f**) The difference between PC1 values of each individual behaviour from day 3 to day 4 indicates the degree of change between the days.

We used a principal component analysis of intruder behaviours from all interactions (days 1–5) to classify behaviours as either proactive or reactive (Fig. 3a). Component 1 (PC1) explained 28.777% of the data and was primarily loaded by resident aggressive behaviours. Component 2 explained 14.027% of the variance and was primarily loaded by intruder behaviours. Intruder behaviours with a positive loading value were classified as proactive (e.g. searching, flee to search) while those with negative loading values were classified as reactive (e.g. hiding/freezing, flinch).

Use of proactive and reactive behaviours changed over the course of repeated social defeat (Fig. 3b; RM ANOVA; proactive: *f*_45,4,157_ = 3.762, *p* = 0.007; reactive: *f*_45,4,157_ = 3.949, *p* = 0.005). Fish initially perform more reactive behaviours than proactive behaviours (*p* = 0.004). By day 3, however, they perform more proactive behaviours than reactive behaviours (*p* < 0.001), but this rapidly reverses on day 4 (*p* < 0.001). To further visualize this shift in behaviours, we used a PCA on intruder behaviours from days 3 and 4 of repeated defeat. Both PCAs produced 1 significant component that explained 35.587% and 27.078% of the variation, respective of days 3 and 4 (Fig. 3d,e). During the 3^rd^ social defeat, proactive behaviours, such as searching, positively loaded onto PC1; however, this flipped during the fourth interaction with reactive behaviours positively loading PC1. Since PC1 seems to correspond to use of each individual behaviour, measuring the difference in PC1 coordinate values between days 3 and 4 of repeated defeat indicates which behaviours increased or decreased and to what magnitude (Fig. 3f). While aggressive score stayed relatively consistent, all proactive behaviours decreased and all reactive behaviours increased while the intruders’ aggression stayed relatively constant. In summary, fish initially perform reactive behaviours, but switch to proactive during the third day of repeated social defeat from the same resident. This dramatic increase in proactive behaviours on day 3 was relatively consistent across all fish (Fig. 3c). Only 5.41% of fish had their most proactive day on day 1. In contrast, 18.92% were most proactive on day 2, 64.86% on day 3, but only 10.81% on day 4. Because of the switch between proactive and reactive coping behaviours across days, fish could not be classified as having a distinct coping style.

Importantly, resident behaviours did not significantly differ across days (RM ANOVA, *f* = 0.946, *p* = 0.441). Although there was variation in resident aggressive scores (number of aggressive behaviours divided by trial time; 4.029 ± 0.328; min = 1.08; max = 9.22), the residents’ aggressiveness did not predict the intruders’ use of proactive and reactive coping behaviours.

### Brain Activation Patterns

**Figure 4:**
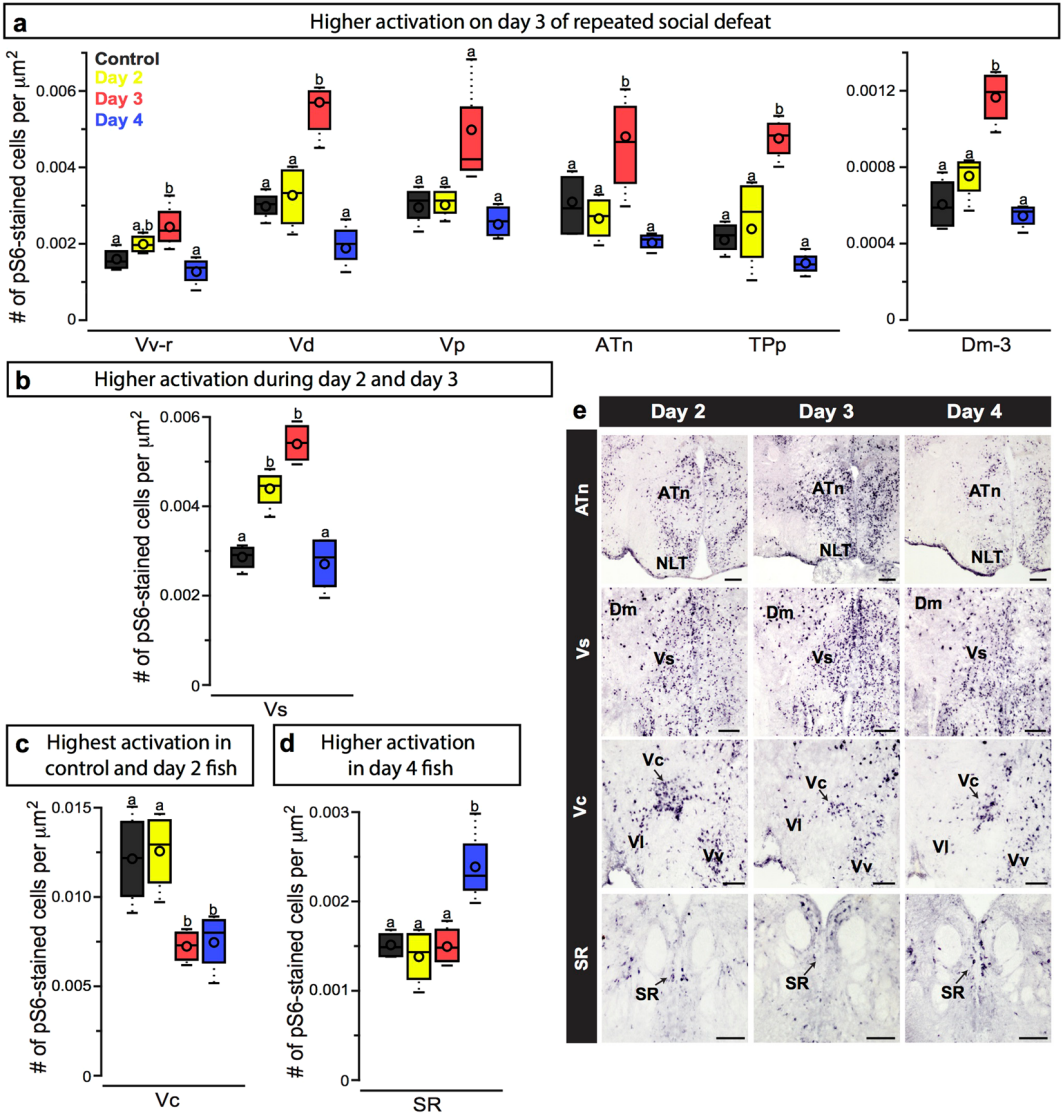
Node by node approach to examine neural activation levels in fish displaying different coping behaviours. (**a**) pS6-stained cell density (i.e. neural activation) in the Vv-r, Vd, Vp, ATn, TPp, and Dm-3 was highest in fish collected after 3 days of repeated social defeat (red). (**b**) In the Vs, fish collected after day 2 (yellow) and day 3 had higher neural activation than control (grey) and day 4 (blue) fish. (**c**) Control fish and those collected on day-2 had higher activation in the Vc compared to fish collected after 3 and 4 bouts of defeat. (**d**) Neural activation in the SR was higher in fish collected after 4 days of defeat when compared with those collected after only 2 or 3 days of defeat. (**e**) Representative photomicrographs of pS6 staining in the ATn, Vs, Vc, and SR from fish collected after 2 (left), 3 (middle), and 4 (right) days of repeated social defeat. Tukey’s box plots were used to plot the data: median is represented by a line and mean by an open circle within the box, the box extends to the furthest data points within the 25^th^ and 75^th^ percentile, and whiskers extend to the furthest data points not considered outliers. Different letters represent statistical significance at *P* < 0.05. N = 4 for all groups. See text for abbreviations. Scale bars in E represent 100 μm for the ATn and Vs panels and 50 μm for the Vc and SR panels.

We compared neural activation patterns in brains collected from different groups of fish after 2, 3, and 4 days of repeated social defeat. Animals used for brain analysis were verified to match the same overall behaviour patterns described above. Of the 14 brain regions examined, fish collected on day 3 had greater activation in six regions (Fig. 4a) compared to fish collected on day 4. In the rostral portion of the ventral part of the ventral telencephalon (Vv-r), there was an overall effect of collection day (*f*_3,15_ = 6.154; *p* = 0.009). Fish collected on day 3 had higher activation than control (*p* = 0.050) and day 4 fish (*p* = 0.008). Fish collected on the 3^rd^ day of repeated social defeat also had higher activation in the dorsal part of the ventral telencephalon (Vd; *f*_3,15_ = 21.458; *p* < 0.001), medial part of the dorsal telencephalon (Dm-3; *f*_3,15_ = 21.648; *p* < 0.001), periventricular nucleus of the posterior tuberculum (TPp; *f*_3,15_ = 14.204; *p* = 0.002), and anterior tuberal nucleus (ATn; *f*_3,14_ = 12.342; *p* < 0.001) than control fish and those collected on days 2 and 4, but there were no differences between these three groups (see Table 1 for post-hoc results). In the supracommissural nucleus of the ventral telencephalon (Vs), fish collected on days 2 and 3 of repeated social defeat had greater activation than control and day 4 fish (Fig. 4b; *f*_3,14_ = 27.557; *p* < 0.001). Although there was an overall effect of the number of defeat interactions (i.e. collection day) on activation in the postcommissural nucleus of the ventral telencephalon (Vp; *f*_3,15_ = 3.899; *p* = 0.037), there were no post-hoc differences.

**Table 1:** Effect of coping behaviours on neural activation measured via pS6-stained cell density.

Region	df	F	P	Tukey’s post-hoc p-values					
				C vs 2	C vs 3	C vs 4	2 vs 3	2 vs 4	3 vs 4
Vv-r	3,15	6.154	**0.009**	0.549	**0.050**	0.697	0.410	0.120	**0.008**
Vv-c	3,15	2.318	0.127						
Vd	3,15	21.458	<**0.001**	0.960	<**0.001**	0.157	**0.002**	0.070	<**0.001**
Vs	3,14	27.557	<**0.001**	**0.009**	<**0.001**	0.979	0.069	**0.005**	<**0.001**
Vc	3,15	14.745	<**0.001**	0.761	**0.004**	**0.009**	<**0.001**	**0.002**	0.994
Vp	3,15	3.899	**0.037**	1.00	0.072	0.998	0.086	0.991	0.053
Dc-4	3,15	1.284	0.324						
Dc-5	3,15	0.382	0.768						
Dm-3	3,15	21.648	<**0.001**	0.364	<**0.001**	0.891	**0.002**	0.127	<**0.001**
Dl-v2	3,13	0.983	0.439						
nPPa	3,15	1.314	0.315						
TPp	3,15	14.204	<**0.001**	0.970	**0.003**	0.532	**0.002**	0.784	<**0.001**
ATn	3,14	12.432	<**0.001**	0.855	**0.003**	0.558	**0.011**	0.202	<**0.001**
SR	3,15	4.611	**0.016**	1.000	0.097	0.051	0.989	**0.041**	**0.022**

Bold indicates statistical significance at *P* < 0.05. C: control; 2, 3, and 4: fish collected on days 2, 3, or 4 of repeated acute social defeat. See text for abbreviations of brain regions.

Control and day 2 fish had higher activation in the central part of the ventral telencephalon (Fig. 4c; Vc; *f*_3,15_ = 14.745; *p* < 0.001) compared to fish collected on days 3 and 4 of repeated social defeat. In the superior raphe (SR), day 4 fish had higher neural activation than control fish and those collected on days 2 and 3 of repeated defeat (Fig. 4d; *f*_3,15_ = 4.611; *p* = 0.016).

There were no differences in the caudal portion of the ventral part of the ventral telencephalon (Vv-c; *f*_3,15_ = 2.318; *p* = 0.127), central part of the dorsal telencephalon (Dc-4: *f*_3,15_ = 1.284; *p* = 0.324; Dc-5: *f*_3,15_ = 0.382; *p* = 0.768), ventral portion of the lateral part of the dorsal telencephalon (Dl-v2; *f*_3,13_ = 0.983; *p* = 0.439), or preoptic area (POA; *f*_3,15_ = 1.314; *p* = 0.315) among all fish groups.

**Figure 5:**
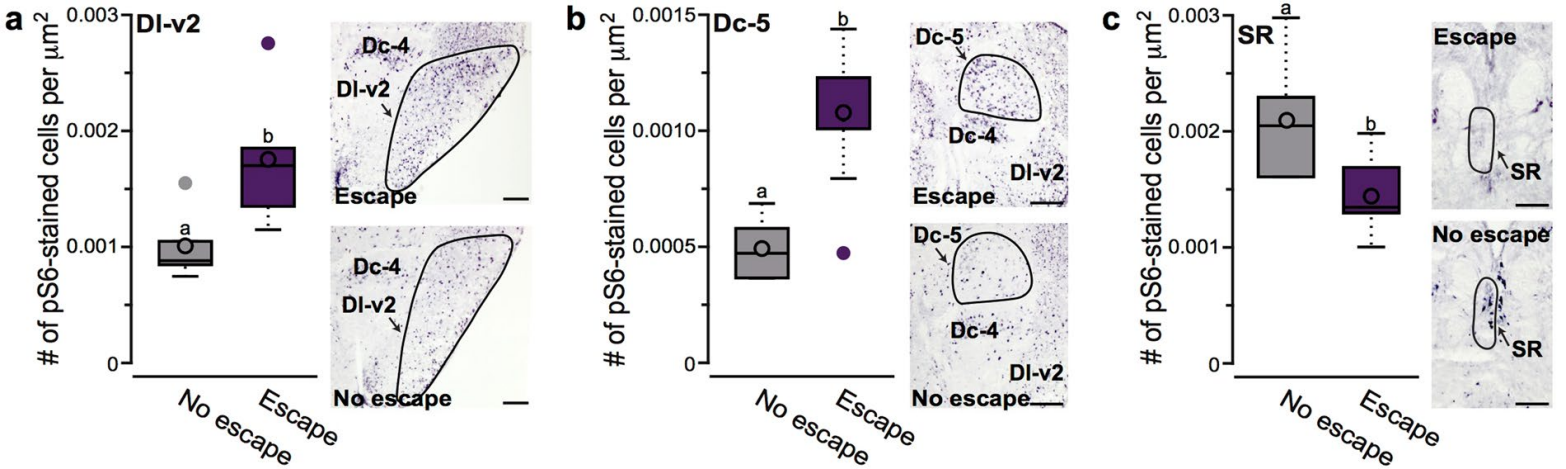
Escaping and non-escaping fish have different patterns of neural activation independent of collection day. (**a**,**b**) Escaping fish have higher activation in Dl-v2 and Dc-5 than non-escaping fish. (**c**) Non-escaping fish have higher activation in the SR compared to escaping fish. See Fig. 4 for box plot descriptions. Different letters represent statistical significance at *P* < 0.05. See text for abbreviations. Scale bars in a and b represent 100 μm and 50 μm in c.

We also examined how escaping through the hole impacted neural activation in all brain regions. There was no interaction between number of social defeats (i.e. collection day) and escape in any region. In both the Dc-5 and Dl-v2, fish who escaped had higher neural activation than those that did not (Fig. 5a,b; Dc-5: *df* = 10, *t* = 2.273, *p* = 0.046; Dl-v2: *df* = 9, *t* = 2.529, *p* = 0.032), but there was no difference in activation due to collection day. However, fish that did not escape had higher activation in the SR than those that did escape (Fig. 5c; *df* = 10, *t* = −2.460, *p* = 0.034).

**Figure 6:**
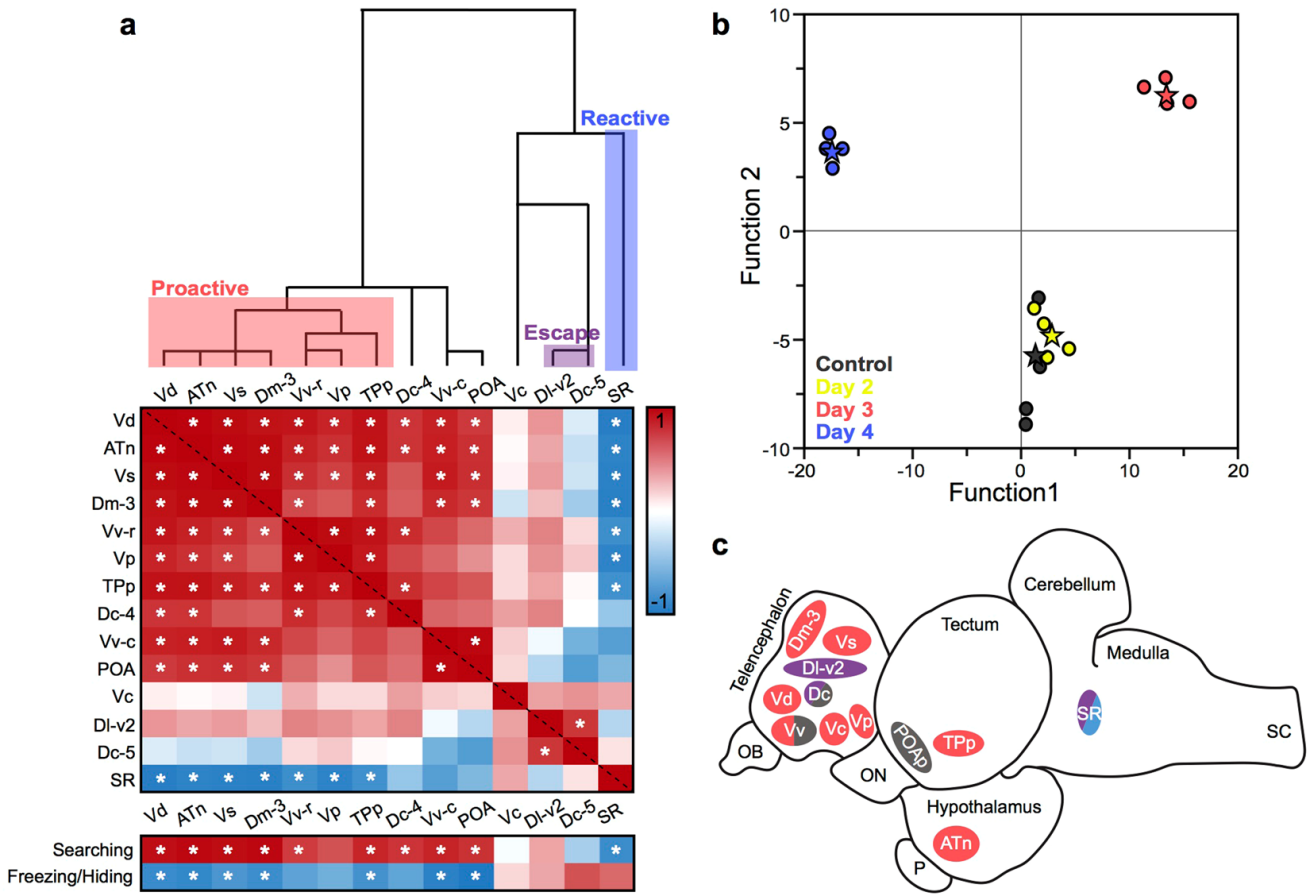
Fish displaying proactive and reactive coping behaviours have distinct patterns of neural activation. (**a**) Hierarchical clustering was used to group brain regions, and Pearson correlation coefficients were used to create a heatmap of neural co-activation across all regions examined, along with searching and freezing behaviours. R-values are represented by colour with red being positive correlations and blue representing negative correlations. Significant correlations (*P* < 0.05) are signified with an*. Boxes designating “proactive”, “reactive”, and “escape” are based on neural activation data in Figs 4 and 5. (**b**) Discriminant function analysis of pS6-staining in all brain regions clearly separates fish displaying proactive and reactive coping behaviours from neutral and control fish, indicating distinct neural activation patterns associated with these opposing coping behaviours. Each animal is represented by a circle and group means by a star. (**c**) Schematic summarizing neural activation results in all regions. Nuclei locations and sizes are approximate. Red represents regions associated with proactive, while blue signifies reactive coping behaviours. Purple represents regions that were different in escaping and non-escaping fish, likely relating to spatial learning. See text for abbreviations.

To determine functional connectivity of nodes in the brain of fish collected after different amounts of social defeat and using different coping behaviours, we generated a heatmap based on Pearson correlation coefficients (Fig. 6a; see Table 2 for statistical values). Regions with greater activation in day 3 fish (i.e. those displaying the most proactive behaviours), the Vd, ATn, Vs, Dm-3, Vv-r, Vp, and TPp, all strongly positively correlated with each other but negatively correlated with the SR. In addition, the Dc-5 and Dl-v2, both regions with greater activation in escaping fish, positively correlated with each other. Further, activation in the Vd, ATn, Vs, Dm-3, Vv-r, Vp, and TPp all positively correlated with searching behaviour but negatively correlate with hiding/freezing behaviours. Conversely, the SR and Dc-5 negatively correlate with searching behaviour but positively correlate with hiding/freezing behaviours. A discriminant function analysis of brain activation across all 14 regions from all fish clearly separates each fish by collection day (Fig. 6b). Function 1 separates fish collected on day 3 and 4, with those collected on day 2 falling in the middle. This corresponds to day 3 fish primarily performing proactive coping behaviours while day 4 fish used predominately reactive coping. Day 2 fish tended to perform approximately equal amounts of proactive and reactive behaviours, and thus fall intermediate between the two. Function 1 had a strong positive loading value from the ATn, Vd, Vs, and Vv-r, but negative loading value from the SR. Interestingly, function 2 separates fish collected on days 3 and 4 from control fish and those collected on day 2. Function 2 had strong positive loading from Dm-3, TPp, Vd, SR, Vp, and Vs, but negative loading by Vc and POA. With both functions, the control and day 2 fish group together. Together, these data suggest a distinct circuit whose activation regulates the use of proactive and reactive coping behaviours.

**Table 2:** Pearson correlation values of pS6 staining in each brain nucleus correlated with all other regions.

Region		ATn	Vs	Dm-3	Vv-r	Vp	TPp	Dc-4	Vv-c	nPPa	Dl-v2	Vc	Dc-5	SR
Vd	R P	**0.982** <**0.001**	**0.956** <**0.001**	**0.918** **0.001**	**0.905** **0.002**	**0.828** **0.011**	**0.913** **0.002**	**0.754** **0.031**	**0.841** **0.009**	**0.737** **0.037**	0.380 0.401	0.077 0.857	−0.143 0.735	**−0.828** **0.011**
ATn	R P		**0.970** <**0.001**	**0.953** <**0.001**	**0.843** **0.009**	**0.776** **0.024**	**0.918** **0.001**	**0.760** **0.029**	**0.853** **0.007**	**0.775** **0.024**	0.325 0.477	0.035 0.934	−0.214 0.611	**−0.797** **0.018**
Vs	R P			**0.95** <**0.001**	**0.781** **0.022**	0.704 0.051	**0.849** **0.008**	0.622 0.100	**0.870** **0.005**	**0.778** **0.023**	0.360 0.428	0.062 0.995	−0.192 0.648	**−0.806** **0.016**
Dm-3	R P				0.670 0.069	0.611 0.107	**0.793** **0.019**	0.596 0.119	**0.805** **0.016**	**0.744** **0.034**	0.250 0.588	−0.192 0.648	−0.339 0.411	**−0.816** **0.013**
Vv-r	R P					**0.954** <**0.001**	**0.911** **0.002**	**0.793** **0.019**	0.682 0.062	0.544 0.163	0.454 0.306	0.298 0.474	0.142 0.737	**−0.725** **0.042**
Vp	R P						**0.886** **0.003**	0.682 0.062	0.562 0.147	0.401 0.325	0.398 0.377	0.144 0.735	0.207 0.622	**−0.792** **0.020**
TPp	R P							**0.788** **0.020**	0.693 0.057	0.619 0.102	0.330 0.469	0.142 0.738	0.043 0.920	**−0.710** **0.049**
Dc-4	R P								0.567 0.143	0.522 0.185	0.451 0.310	0.346 0.401	<0.001 1.00	−0.374 0.361
Vv-c	R P									**0.965** <**0.001**	−0.056 0.905	0.187 0.658	−0.506 0.201	−0.625 0.098
nPPa	R P										−0.271 0.556	0.153 0.717	−0.636 0.090	−0.463 0.248
Dl-v2	R P											0.341 0.455	0.743 0.056	−0.273 0.554
Vc	R P												0.395 0.333	0.318 0.442
Dc-5	R P													0.117 0.782

Pearson correlation coefficients (*R*) were used to create the heatmap in Fig. 6a. Bold indicates significance at *P* < 0.050.

## Discussion

We show for the first time in fishes that use of proactive and reactive coping behaviour changes over time in the same individuals exposed to repeated acute social defeat, but that fish do not appear to adopt a single “coping style”. During the first two social defeat interactions, individual fish used both proactive and reactive coping behaviours. However, fish increased their use of proactive behaviours while simultaneously decreasing use of reactive behaviours during subsequent interactions. All fish reached a threshold, most commonly on the third interaction, where their proactive coping behaviours peaked. The subsequent day had a drastic reduction in proactive, but increase in reactive behaviours. This timeline is novel, and suggests that coping behaviours may not be as predictable and stable as previously thought.

Social defeat provides an excellent paradigm to study coping behaviours in a naturalistic way. Animals that live in social communities, as in the natural lek-ing environment of *A. burtoni*, are constantly exposed to social rank dynamics. They encounter the same individuals on a regular basis. Animals of lower rank may be chronically (continually, no chance to rise in rank) or repeatedly (intermittent, rise in rank between each defeat) defeated, and it is important to understand how these repeated interactions affect coping behaviours and ultimately survival and reproductive fitness. Since subordinate individuals often experience chronic social defeat, these behaviours and neural data may only generalize to dominant individuals suffering repeated acute defeat. Fish in our experiments suffered repeated acute social defeat by the same resident aggressor, as they returned to territoriality in their home compartments between daily trials. This provided an opportunity to study the neural correlates of social defeat in an ecologically-relevant setting. It is important to note that not all fish responded to the resident-intruder paradigm in a measurable way. Approximately 12% of the experimental fish were removed from the data analysis because they failed to perform a single behaviour during any of the daily trials. Similarly, Wood *et al*. (2011) identified a subset of male *A. burtoni* that failed to perform in a spatial learning paradigm, classifying ~8% of their subject males as non-attempters^51^. While the behavioural paradigms are very different, both studies found a similar percentage of fish that lacked a response to the behavioural paradigms. Wood *et al*. 2011 found that non-attempting fish were characterized by high levels of cortisol. These non-responding fish in our study could reflect an ultimate form of reactive coping, but were not collected in our study because our focus here was on the switch from proactive to reactive coping during repeated interactions.

The majority of experimental fish showed an increase in proactive coping behaviours during the third interaction, however, there was some individual variation with the most proactive trial being on day 2 or 4 in ~30% of fish. All fish did eventually reach some threshold after which they switched to using reactive coping behaviours, but the timing varied among individuals. Unfortunately, we were unable to identify factors contributing to this variability, but individual variation in behaviours is common in social animals. It is possible that the individual variation is due to differences in physiology. Stress response and HPA reactivity is highly variable in *A. burtoni* males^49,52^, so this threshold may be dictated by their innate stress physiology. In addition, recent research suggests that there are several types of dominant males, and that these males have different behavioural and hormonal profiles^53^. This variation in HPA activity and differences in hormonal profiles may determine when an individual reaches its threshold and switches from proactive to reactive coping.

Fish displaying proactive behaviours (i.e. those collected on day 3) had higher neural activation in several socially-relevant brain regions. In the Vd, rostral Vv, ATn, TPp, and Dm-3, fish had greater activation only on day 3 of repeated social defeat. All of these regions are implicated in regulating or responding to social defeat or other stressors in both fishes and mammals^7,9,51,54–56^. For example, *A. burtoni* males descending in social status have higher levels of immediate early genes (*cfos*, *egr1*) in Dm, Vv, Vs, and ATn than non-descending males^30^. In addition, Atlantic Salmon (*Salmo salar* L.) displaying proactive behaviours in response to an environmental stressor (hypoxia) had more serotonin receptors (i.e. *5-HT*_*1Aα*_, *5-HT*_*1Aβ*_) in Dm and Vv than reactive coping fish^9^. Hamsters also have an increase in immediate early gene expression in the lateral septum (mammalian homolog of Vv, in part) and ventromedial hypothalamus (mammalian homolog of ATn, in part) following social defeat^55^, but interestingly, Martinez *et al*. (1998) found greater *cfos* expression in the lateral septum only in response to a single social defeat but not during subsequent defeats in male rats^56^. In the bed nucleus of the stria terminalis and medial amygdala, the putative homologs of the teleost Vs, male rats had increased *cfos* expression after both single and repeated social defeat^56^. Similarly, *A. burtoni* males collected on days 2 and 3 of repeated social defeat had greater pS6 staining in the Vs. Interestingly, in our study, increased neural activation in the Vs was greatly reduced after the fourth defeat interaction, during which the fish behaved primarily with reactive coping behaviours. It is important to note that in these regions (i.e. Dm-3, Vv-r, Vd, ATn, TPp), animals collected on days 2 and 4 of repeated defeat had neural activation patterns similar to asocial control animals. Thus, the greater activation seems specific to the increase in proactive coping behaviours observed on day 3, as further evidenced by the high degree of positive correlations between activation in Vv-r, Vd, ATn, TPp, Dm-3, and Vs with searching behaviours.

We also observed greater activation in the TPp of fish displaying proactive behaviours (i.e. day-3 fish) compared to those fish displaying reactive behaviours collected on days 2 and 4. In chronically-defeated mice, the firing rate of ventral tegmental area (putative homolog to the teleost TPp) neurons negatively correlates with social avoidance (i.e. reactive coping behaviour)^54^. Similarly, we found that TPp activation was lowest in animals displaying reactive behaviours, and activation negatively correlated with “freezing/hiding” behaviours (i.e. social avoidance). The mammalian VTA and teleost TPp contain large dopaminergic cell populations and are likely key regulators of the mesolimbic reward system^54,57^. The mesolimbic reward system is known to underlie susceptibility and resistance to social defeat in rodents, primarily through the connections of the VTA to the nucleus accumbens (NAcc)^54,58^. As both the TPp and Vd (putative teleost homolog of NAcc) have greater activation in proactive fish, and this activation positively correlates with searching behaviours, further studies should examine the potential importance of dopamine signalling within the mesolimbic reward system that may be related to fish coping behaviours.

Although we identified several brain regions that corresponded to proactive coping seen on day 3 of repeated defeat, we also identified two regions related to neutral and reactive coping observed on days 2 and 4, respectively. We found that fish collected after 2 defeat interactions had higher activation in Vc than fish collected after 3 and 4 bouts of defeat. The Vc contains a large population of corticotropin-releasing-factor (CRF) cells in *A. burton*i^49^. As day 2 fish had similar levels of activation to the asocial, control fish, it is possible that the greater activation in Vc is a reaction to the handling stress or novel environment. Proactive- and reactive-behaving fish, collected on days 3 and 4 of repeated defeat, may have acclimated to the stress of the paradigm by this point, resulting in lower levels of activation. Fish displaying increased reactive coping behaviours (i.e. collected on day 4) had higher activation in the superior raphe, but no other region. Importantly, the SR contains a large population of serotonin cells^59^. Across taxa, animals displaying reactive coping behaviours have higher levels of serotonin in the brain, including in the raphe^2,60–64^. As this is the only region we examined with greater activation in reactive-behaving fish, we propose that this region may in part mediate the expression of reactive behaviours in *A. burtoni*.

Our experimental design offered fish an “escape hole”, which allowed the intruder to completely escape from the resident and return to his home territory. Escaping from a stressor is commonly used to measure proactive-reactive behaviours in fishes. We identified two regions of the brain, the Dl-v2 and Dc-5, that had greater activation in fish that escaped over those that did not, independent of the types of coping behaviours used or the number of social defeats. The Dl, putative homolog to the mammalian hippocampus, is involved in social and spatial learning and memory in fishes. Lesions to the Dl in goldfish reduces their ability to perform spatial tasks^65,66^. The higher activation of Dl in escaping fish likely reflects its involvement in searching for and/or identifying the location of the escape hole. Although homology of the teleost Dc is not well established, some research has suggested it is homologous in part to the globus pallidus (GP)^67^. Because the GP is involved in regulating voluntary movements, it is possible that more activity in Dc may be related to the act of searching and/or swimming through the escape hole. Searching time was variable across individuals and did not necessarily predict whether an animal escaped or not. This greater neural activation, therefore, likely does not reflect the increased searching but is more related to the directed, location-sensitive behaviours of identifying the escape hole. Others have suggested that the Dc, in part, may represent deep layers of the cerebral cortex^68,69^. Elliot *et al*. (2017) also demonstrated strong connections from the Dl to the Dc^68^. Finally, we found that animals not escaping through the hole had higher activation in SR than those that did escape. Similarly, Carpenter and Summers (2009) found that non-escaping rainbow trout had higher levels of dopamine and serotonin in the raphe than those that did escape^6^. We expected that the regions preferentially activated in escaping fish (i.e. the Dl-v2 and Dc-5) would cluster with proactive behaviours, as escaping is more of a proactive-like behaviour and grouped strongly with other proactive behaviours in our PCA. Instead, the Dl-v2 and Dc-5 both grouped more closely with the SR. This is likely due to the difference in the SR activation between escaping and non-escaping fish and does not reflect a functional connection. Further, activation of the Dc-5 and Dl-v2 positively correlate with each other but not with SR activation. We suggest that escaping does not necessarily reflect a coping style, but rather spatial awareness and learning. In our experiments, escaping or not was not reflected by activation in the functional network regulating proactive behaviours.

We identified a group of brain regions with greater neural activation only when fish are using higher levels of proactive coping behaviours, and whose activity strongly correlates with each other and with freezing and searching behaviours. In addition, hierarchical clustering grouped these regions together, and they all strongly loaded function 1 of the DFA, which clearly separated proactive-behaving (day 3) and reactive-behaving (day 4) fish based on brain activation alone. Further, activation of this network was strongly negatively correlated with activation in the SR, which was also separated in the hierarchical clustering and was the strongest predictive factor in reactive coping fish (i.e. collected on day 4). Together, these data suggest a possible connection between the neural network in the diencephalon regulating proactive coping behaviours and the superior raphe of the hindbrain. Many of the examined brain regions contain serotonin receptors^9,59^. Since greater activation in the SR suggests increased serotonin production/metabolism, we propose that the SR, through serotonin signalling, may be one of the key mediators of proactive vs reactive coping. Similarly, research across taxa has found that serotonin, its metabolite, and its receptors are differentially expressed between animals displaying proactive and reactive coping^70^. In *A. burtoni*, differences in the serotonin signalling pathway lead to differences in escape behaviours via modulation of Mauthner cell activity^71^. In addition, the suite of other neuropeptides (e.g. CRF, urotensin) and neurotransmitters (e.g. dopamine) shown to be important for proactive and reactive coping can interact with serotonin signalling to ultimately regulate these coping behaviours^9,18,58,62^. As many of the examined regions contain CRF- and urotensin-producing cells and/or CRF receptors^49,72^, it is likely that there is interplay between these systems. In our experiment, fish using similar levels of proactive and reactive behaviours had greater activation in the Vc compared to those predominately using proactive or reactive coping behaviours. Because the Vc contains a large population of CRF-producing cells, and many of the brain regions correlating to proactive behaviours contain CRF receptors^49,72^, perhaps increased CRF signalling modifies the network that typically produces proactive behaviours. Similarly, without higher activation of the SR, and therefore increased serotonin output, there is no overt increase in reactive coping behaviours. However, reduced CRF signalling on day 3 may allow for activation of this functional network and the display of proactive coping behaviours. Finally, fish with greater activation in the SR and potentially more serotonin output, may inhibit the “proactive” network and promote the display of reactive coping behaviours. Further research combining our node-by-node approach with monoamine and neuropeptide measures are needed to fully understand the neural networks regulating proactive and reactive coping.

Fishes are increasingly used as models for understanding human mental health disorders, such as depression and anxiety^16^. For this comparative and translational approach to be effective, it is important to understand the evolution and conservation of neural networks underlying the behaviours typically displayed in these neurological conditions. We provide a node-by-node approach to investigating the regulation of proactive and reactive coping behaviours. By combining this with previous studies on monoamine neurochemistry and neuropeptide and receptor abundance, we can begin to untangle the functional homologies of fish brain nuclei, an important step towards validating fishes as model systems for future research. We hope that these data will spur more functional studies to understand how these regions may regulate proactive and reactive coping behaviours across vertebrates.

## Electronic supplementary material

Supplementary Information
