# Supplementary material for: Neural and behavioural correlates of repeated social defeat
Source: Sci Rep. 2018 May 1;8:6818. doi: 10.1038/s41598-018-25160-x (PMC5931592; doi:10.1038/s41598-018-25160-x)
Supplement: Supplementary file 1 — Supplementary Information [file 41598_2018_25160_MOESM1_ESM.pdf]

Neural and behavioural correlates of repeated social defeat

Julie M. Butler*, Sarah M. Whitlow, David A. Roberts, and Karen P. Maruska

Behavior	Definition
Chase	One fish chases other fish around tank; lasted minimum of two seconds
Lunge	Fish distends jaw and flares opercula; often accompanied by a lunge at another fish (similar to a Frontal Display)
Nudge/Bite/Ram	One fish rams opponent typically on the trunk with an open mouth (bite) or closed mouth (nudge)
Lateral Display	Fish orient parallel to each other, erect fins, distend jaws, and shake their bodies
Total Aggressive Behaviors	Sum of 'chase', 'lunge', 'nudge/bite/ram', and 'lateral display'
Aggressive Score	Total number of aggressive behaviors divided by the length of the trial
Hiding Time	Time fish spent stationary in the bottom corner of the tank or top of the water column; lasted minimum of two seconds
Searching Time	Time fish spent swimming perpendicular into the wall of the tank or barrier, often in the vertical plane; lasted a minimum of two seconds
Correct Searching Time	Time spent "searching" (see above) on the barrier that contained the escape hole
Other Searching Time	Time spent "searching" (see above) on a wall that did not contain the escape hole
No Movement	In response to resident aggressive behavior, intruder does not move
Flinch	In response to resident aggressive behavior, intruder moved within 2 body length away or changed orientation
Flee to Search	In response to resident aggressive behavior, intruder leaves current location (flee) and performs a 'searching' behavior within 2 seconds
Flee to Hide	In response to resident aggressive behavior, intruder leaves current location (flee) and performs a 'hiding' behavior within 2 seconds
Flee to Other	In response to resident aggressive behavior, intruder leaves current location (flee) but does not perform either 'searching' or 'hiding' behaviors
% Hiding Time	Total time hiding divided by the length of the trial
% Other Searching	Total time 'other searching' divided by the length of the trial
% Correct Searching	Total time 'correct searching' divided by the length of the trial
Total searching %	Sum of ' % other searching' and ' % correct searching'
No Move %	Number of 'No Movements' divided by the total # of aggressive behaviors by the resident
Flinch %	Number of 'Flinch' divided by the total # of aggressive behaviors by the resident
Flee/Search %	Number of 'Flee to Search' divided by the total # of aggressive behaviors by the resident
Flee/Hide %	Number of 'Flee to Hide' divided by the total # of aggressive behaviors by the resident
Flee w/other %	Number of 'Flee to Other' divided by the total # of aggressive behaviors by the resident
